# Engineering conformational transitions in silk fibroin hydrogels to create advanced dynamic microenvironments for biomedical applications

**DOI:** 10.1093/rb/rbag089

**Published:** 2026-05-07

**Authors:** Xiang Yao, Xueqian Xu, Guolong Cai, Weikun Zhao, Wanqin Yao, Suna Fan, Qianqian Niu, Yaopeng Zhang

**Affiliations:** State Key Laboratory of Advanced Fiber Materials, College of Materials Science and Engineering, Shanghai Engineering Research Center of Nano-Biomaterials and Regenerative Medicine, Donghua University, Shanghai 201620, China; State Key Laboratory of Advanced Fiber Materials, College of Materials Science and Engineering, Shanghai Engineering Research Center of Nano-Biomaterials and Regenerative Medicine, Donghua University, Shanghai 201620, China; State Key Laboratory of Advanced Fiber Materials, College of Materials Science and Engineering, Shanghai Engineering Research Center of Nano-Biomaterials and Regenerative Medicine, Donghua University, Shanghai 201620, China; State Key Laboratory of Advanced Fiber Materials, College of Materials Science and Engineering, Shanghai Engineering Research Center of Nano-Biomaterials and Regenerative Medicine, Donghua University, Shanghai 201620, China; State Key Laboratory of Advanced Fiber Materials, College of Materials Science and Engineering, Shanghai Engineering Research Center of Nano-Biomaterials and Regenerative Medicine, Donghua University, Shanghai 201620, China; State Key Laboratory of Advanced Fiber Materials, College of Materials Science and Engineering, Shanghai Engineering Research Center of Nano-Biomaterials and Regenerative Medicine, Donghua University, Shanghai 201620, China; State Key Laboratory of Advanced Fiber Materials, College of Materials Science and Engineering, Shanghai Engineering Research Center of Nano-Biomaterials and Regenerative Medicine, Donghua University, Shanghai 201620, China; State Key Laboratory of Advanced Fiber Materials, College of Materials Science and Engineering, Shanghai Engineering Research Center of Nano-Biomaterials and Regenerative Medicine, Donghua University, Shanghai 201620, China

**Keywords:** silk fibroin hydrogel, protein conformational transition, dynamic material microenvironment, cartilage tissue engineering, cell-material interaction

## Abstract

Among varied silk fibroin (SF) materials, chemically crosslinked SF hydrogels (SFHs) showed excellent biocompatibility and ECM-mimetic property, thus being widely used in biomedical applications. Intriguingly, recent studies have uncovered a unique dynamic material cue in SFHs—the protein conformational transition microenvironment. This microenvironment induces a dynamic material stiffening/shrinkage process and further significantly regulates cell behaviors, thus presenting another promising dynamic material cue for biomedical applications. Very recently, to enhance the controllability of this microenvironment and elucidate how conformational transition rates influence cell behaviors, novel strategies for regulating the transitions have been developed, leading to deeper insights into the corresponding cell–microenvironment interactions. Focused on this dynamic microenvironment, we seek to comprehensively describe the intrinsic mechanism of the transitions, the dynamic material features induced by the transition and corresponding characterization methods. It also highlights recent findings and advances in the effective regulation strategies, as well as their promising biomedical applications. Finally, current challenges and future prospects regarding the engineering of this unique microenvironment and its potential applications are comprehensively discussed. This review aims to effectively expand the knowledge of dynamic material cues and the related cell-material interactions and also offers valuable insights for the development of unique SF and other protein-based biomaterials.

## Graphical abstract

**Graphical Abstract:**
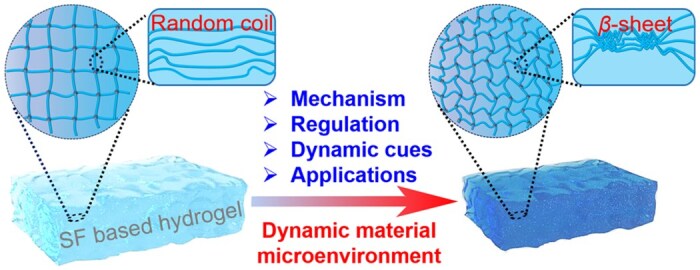

## Introduction

Cells within the organism continuously contact a very complex microenvironment that includes diverse static and dynamic stimulation factors [1–4]. The realization of varied cell functions primarily relies on cells’ responses to these factors, thereby enabling the functionalization of their resident tissues. For instance, cells can respond to soluble factors through specific receptors or channels on the cell membrane, sense various physicochemical features of the extracellular matrix (ECM) and substrate materials via integrin-mediated focal adhesion, such as the chemical composition [5–7], modulus [8, 9], topological structure [10–14] and nanoscale features [11, 15–17]. Additionally, cells can also communicate with other neighboring cells through cadherin-mediated intercellular adhesion and gap junctions formed by connexins [18]. Many specialized functions of the cells and tissues are precisely achieved through the integration of specific cell-microenvironment interactions.

Among various microenvironmental factors, research on the static factors and their effects on cell function regulation and tissue repairing is relatively extensive. Compared with static ones, investigations on the dynamic factors’ impacts on cell behaviors and their effective utilization are relatively insufficient. However, cells *in vivo* are usually exposed to dynamically changing microenvironments, such as the degradation and remodeling of ECM [19], the dynamic shear and circumferential stress generated by blood flow, as well as the dynamic shear, tensile or compressive stress induced by joint movements, etc.

In existing reports on the effects of dynamic microenvironments on cell behavior and tissue repairing, the majority of them focused on the dynamic mechanical stimuli and material degradation cues. The reported dynamic mechanical stimuli mainly contain dynamic hydrostatic pressure, periodic mechanical stretching and fluid shear stress. For example, scientists have proved that hydrostatic pressure stimulation can significantly affect cell adhesion and differentiation, particularly benefiting the phenotype maintenance of chondrocytes and the chondrogenesis of stem cells [20, 21]. Periodic mechanical stretching of cell culture substrates has also been demonstrated to obviously influence cell orientation, proliferation and differentiation [22, 23]. Additionally, proper fluid shear stress stimulation generated by the culture fluid in a cell culture system could significantly enhance stem cell spreading and osteogenesis [24]. As for the dynamic material cues, most studies focused on the material degradation process [25–29]. For instance, scientists have constructed degradable and nondegradable polyethylene glycol hydrogels [30, 31] or hyaluronic acid hydrogels [32] to study how the degradation processes influence cell behaviors. Results demonstrated that stem cells exhibited better spreading and higher mechanical stress in degradable hydrogel systems (relatively softer), which favored osteogenic differentiation. In contrast, cells in nondegradable hydrogel systems (relatively stiffer) showed poorer spreading and weaker stress, being more conducive to adipogenesis [30–32]. These outcomes could not be fully explained by the traditional static substrate stiffness effects on cell fates, where stiffer substrates typically promote cell spreading and osteogenesis, while softer ones enhance adipogenesis [33, 34]. This discrepancy indirectly suggests that the material degradation process itself may serve as a new independent dynamic material microenvironment for regulating cell functions. These valuable findings provided solid evidence for the importance of dynamic microenvironments and crucial guidance for constructing effective tissue engineering scaffolds or bioreactors suitable for enhancing cell expansion and directional differentiation, which also offers important references for the understanding and prevention of relevant diseases.

As a well-known biomacromolecule, silk fibroin (SF) has emerged as an important biomaterial extensively researched and developed due to its comprehensive advantages, including excellent biocompatibility, facile processability, tunable degradation and abundant sources [34–43]. Among different kinds of SF materials, SF hydrogels (SFHs) showed outstanding cytocompatibility and ECM-mimetic properties. The physically crosslinked SFHs exhibit mild formation conditions while suffering from prolonged gelation time and relatively weak mechanical properties [34, 44, 45]. For chemically crosslinked SFHs, excessive crosslinking density could significantly reduce the permeability of nutrients and metabolism products, thereby limiting their biomedical applications. In light of these factors, the development of chemically crosslinked hydrogels featuring appropriate crosslinking densities, fabricated via mild crosslinking strategies, has attracted considerable research interest in recent years [46–50]. Mild chemical crosslinking facilitates the encapsulation of living cells within SFHs, enabling the establishment of an effective three-dimensional (3D) cell culture system. This hydrogel system also permits the encapsulation and sustained release of vulnerable growth factors or drugs. Recent studies have demonstrated promising applications of such mildly crosslinked SFHs in tissue repair and 3D cell culture [25, 37, 51–53].

**Figure 1:**
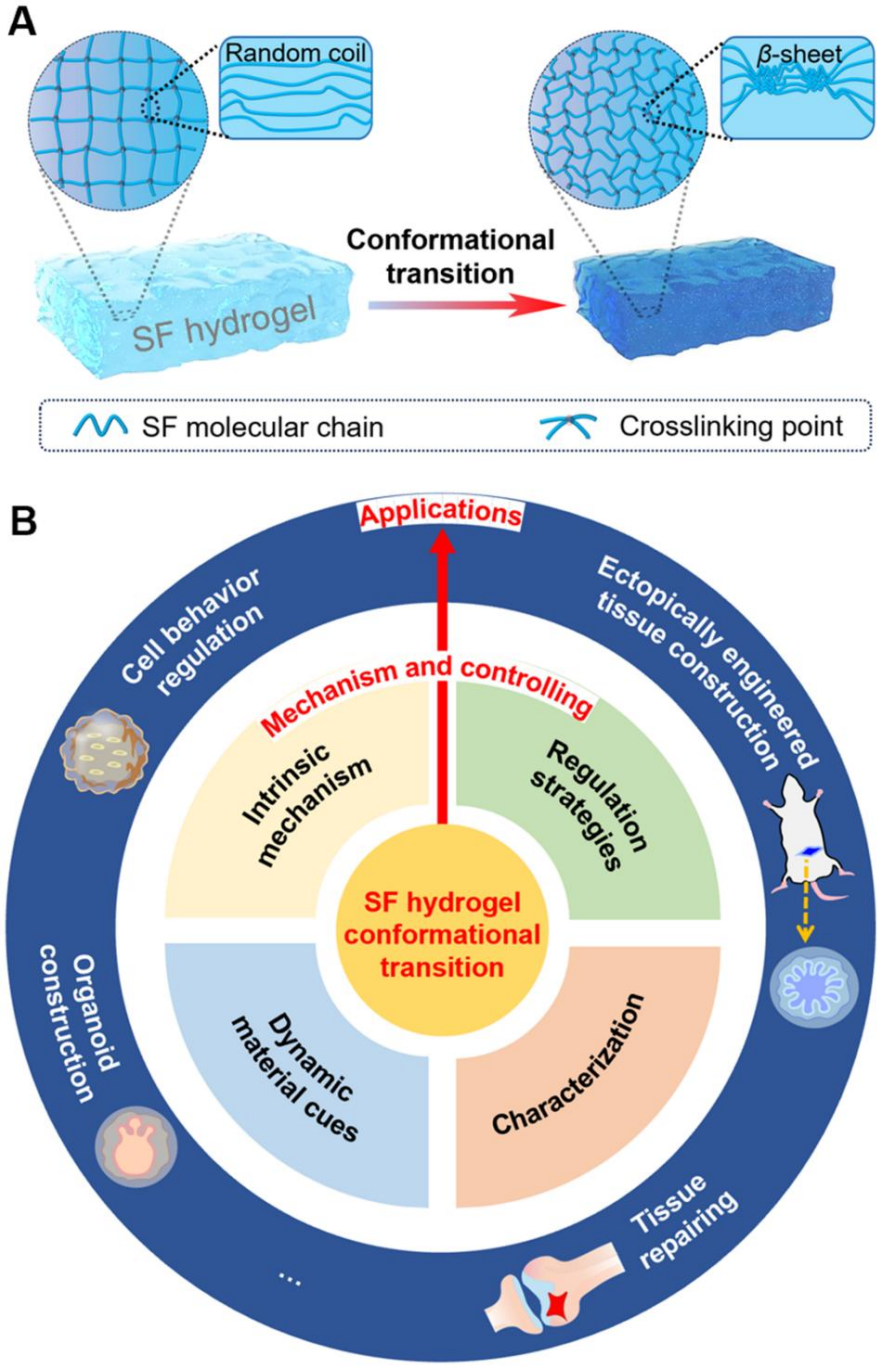
(**A**) Schematic presentation to show the dynamic conformational transition of proteins within SFH; (**B**) summarized outlines to highlight the main contents of this review: the intrinsic mechanism, corresponding regulation strategies and induced dynamic material cues of the CTM and its existing and promising applications. Image (A) was modified with permission [54]. Copyright 2025, Oxford University Press.

Interestingly, some frontier in-depth investigations have revealed a unique dynamic material microenvironment in these SFHs—the protein conformational transition microenvironment. More specifically, under *in vivo* or *in vitro* cell incubation conditions, the dynamic motion and self-assembly of SF hydrophobic segments facilitate the protein conformation shift from random coils to lower-energy *β*-sheet structures [54, 55]. This process, in turn, induces a dynamic transition of protein conformation within the SFH, as outlined in Figure 1A. This dynamic transition process visibly transforms the hydrogel from transparent to opaque, accompanied by alterations in crucial material features, including modulus, pore size and porosity. Unlike the widely studied dynamic degradation of materials, which typically results in a gradual softening and expansion process, the conformational transition of protein in SFHs leads to a dynamically stiffening and shrinking material microenvironment [54, 55]. Although certain adaptive crosslinking hydrogels have been reported to exhibit similar dynamic stiffening and shrinkage to some extent [56], the corresponding material design and synthesis remain relatively complex, and there is still considerable room for improvement in the controllability of the relevant dynamic factors, especially extending its duration of action. Further pertinent studies about the conformational transition microenvironment (CTM) have demonstrated that this dynamical microenvironment in SFHs could be effectively engineered via material design and external stimuli [54, 55, 57–59], and this kind of dynamic material microenvironment could further significantly regulate cell behavior and function [54, 55, 57]. Therefore, the protein conformational transition within SFHs represents another unique dynamic material microenvironment fundamentally different from material degradation, which also holds great application potential in the biomedical field.

Focused on this unique dynamic material microenvironment in SFHs, this review first systematically summarized the commonly used synthesis strategies of SFHs. Then, the intrinsic mechanism of the protein conformational transition, the dynamic material features caused by this transition process, the comprehensive characterization methods and effective adjusting strategies of this transition process were carefully summarized. In addition, we further elaborated the specific application research progress of this dynamic material microenvironment in the fields of stem cell and cancer cell function regulation and the construction of ectopically engineered tissue under an *in vivo* situation. Finally, current challenges and future opportunities for this unique dynamic material microenvironment regulation and application scenarios are also comprehensively discussed, as briefly summarized in Figure 1B. This summarized information effectively expands the core knowledge in the fields of dynamic material cues and the related cell-material interactions, which could provide precious insights for designing novel biomaterials and pave the way for the highly efficient utilization of SF and other similar biomacromolecule-based biomaterials.

## The fabrication of SFH

SF could be used alone or compounded with other polymers to prepare hydrogels. Currently, there are many kinds of SF-based hydrogels that have been developed, which could be generally classified into physically or chemically crosslinked hydrogels based on their crosslinking mechanisms.

In the physical crosslinking strategy, SF molecules are induced to form *β-*sheet structures as the crosslinking points under the stimulation of external environments, thus forming a physical crosslinking network between a huge number of SF molecules. Different from physically crosslinked hydrogels, the formation of covalent bonds as crosslinking points between SF molecules is necessary for the chemically crosslinked hydrogels [34]. Comparably, SF molecules in the chemically crosslinked hydrogels remain much more random coil structures after gelling, so the chemically crosslinked hydrogels usually present a larger pore size and better elasticity and mechanical properties.

### Physical crosslinking strategies

**Figure 2:**
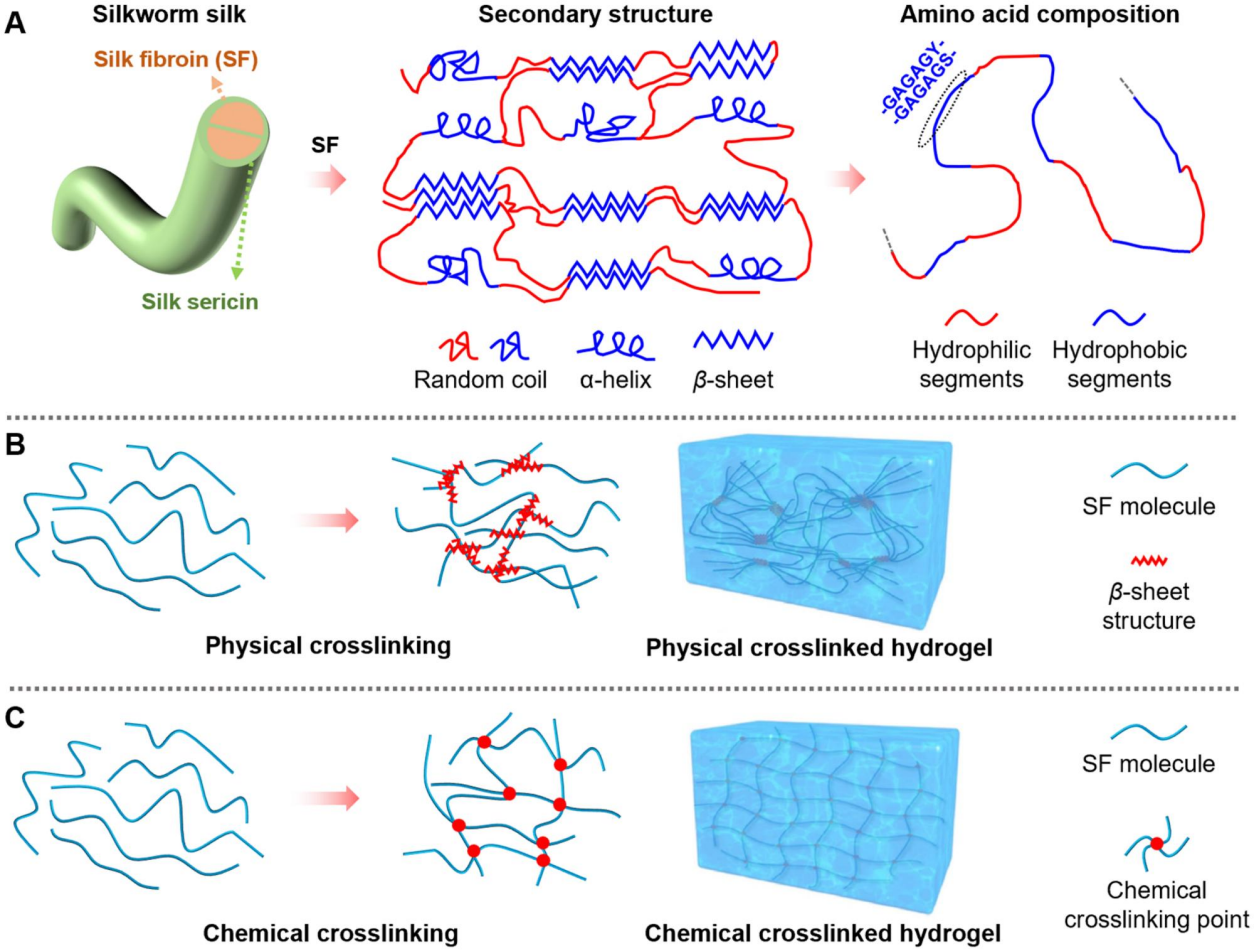
Schematic illustration to show the inherent SF molecule structures and the general formation mechanism of SFHs. (**A**) Inherent molecular features of SF; (**B**) cartoon pictures to show the formation mechanism of SFHs *via* physical crosslinking; (**C**) cartoon pictures to show the formation mechanism of SFHs *via* chemical crosslinking. The cartoon pictures of the indicated hydrogels in images (B) and (C) were reproduced with permission [34].Copyright 2022, Elsevier.

As a natural macromolecule, SF molecule chains mainly consist of alternating hydrophobic and hydrophilic chain segments [36, 60]. The hydrophilic chain segments tend to adopt random coil conformation, whereas the hydrophobic chain segments can feasibly self-assemble into *α*-helix and *β*-sheet conformations with each other [36, 61–63], as schematically shown in Figure 2A. More specifically, the hydrophobic chain segments of SF molecules are primarily characterized by repetitive polypeptide sequences, such as (-GAGAGS-)_n_ and (-GAGAGY-)_n_. These sequences could endow the formation of *β*-sheet structures with high binding energy (see Figure 2A) [34, 36]. Consequently, natural SF possesses a semicrystalline nature, where regular antiparallel *β*-sheet crystals are dispersed within an amorphous matrix [61, 64, 65], thus exhibiting excellent comprehensive mechanical properties.

Just because of these inherent special hydrophobic segments, when SF molecules come into contact with each other, they gradually self-assemble to form the corresponding *β*-sheet structures as physical crosslinking points. And when a physical crosslinking network between a huge number of SF molecules is formed, the physically crosslinked SFH can be formed [34], as shown in Figure 2B.

It has been shown that the formation of *β*-sheet structures in SF solutions could be significantly accelerated by high SF molecule concentration, ultrasound stimulation, shear induction, electrical field stimulation, suitable temperature stimulation, etc. [34]. Thus, these kinds of induction strategies could probably be used for the fabrication of physically crosslinked SFH. For example, Yuan *et al.* [66] have proposed a one-step ultrasonication strategy to fabricate physically crosslinked SFH through an SF aqueous solution. The ultrasound stimulation-induced SFH presented desired physicochemical and biomechanical properties and could promote chondrogenesis in both *in vitro* and *in vivo* conditions. In addition, Yucel *et al.* [67] have found that vortexing aqueous solutions of SF could also lead to the formation of *β*-sheet structures, thus inducing the formation of physical crosslinking points and the corresponding SFH. Further research revealed that the hydrogelation kinetics could be effectively adjusted by altering the vortex time, assembly temperature and/or protein concentration. This strategy could generate SFHs using very simple equipment without any contact probes to the aqueous solution. Under suitable electrical stimulation, Leisk *et al.* [68] have also successfully fabricated physically crosslinked SFHs on the positive electrode based on SF aqueous solution. Furthermore, it was shown that switching the voltage polarity led to the redeposition of the hydrogel, which then began to form on the newly activated anode.

In summary, the core mechanism behind the formation of physically crosslinked SFHs relies precisely on the protein conformational transition, wherein the conformational transition of SF molecules in solution facilitates its transformation from sol to gel state. The formation of physically crosslinked SFHs could also help us better understand how external environmental stimuli induce conformational transitions of SF molecules in the SF solutions.

### Chemical crosslinking strategies

Different from the physical crosslinking strategies, direct covalent bonds between SF molecules form in chemically crosslinked hydrogels. Until now, the corresponding covalent bonds are usually formed by the small molecule crosslinking agents, the phenolic hydroxyl groups between the amino acids of tyrosine in SF molecules or the carbon–carbon double bonds between the modified SF molecules. When a chemical crosslinking network between a huge number of SF molecules is formed, the chemically crosslinked SFH can be obtained [34] (see Figure 2C).

#### Crosslinked by the small molecule crosslinking agents

Generally, the special small molecule crosslinking agents have multiple reactive groups which react with existing functional groups such as -NH_2_, -OH and –COOH in the SF molecules and thus can form a chemically crosslinked network structure. The typically used small crosslinking agents for SFH fabrication are glutaraldehyde and genipin.

**Figure 3:**
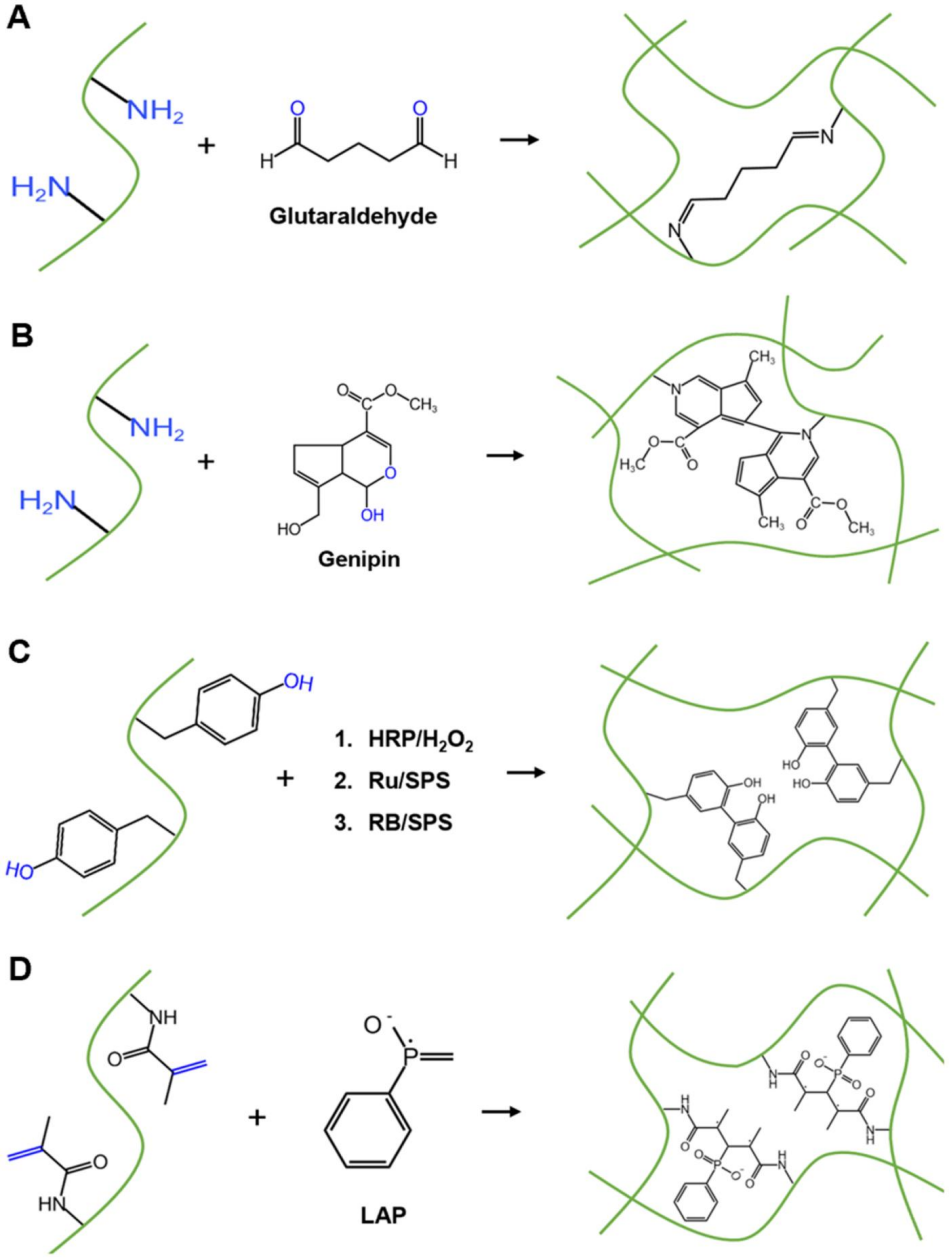
Formation mechanisms of the typical chemically crosslinked SFHs. (**A**) Glutaraldehyde-crosslinked hydrogel; (**B**) genipin-crosslinked hydrogel; (**C**) HRP/H_2_O_2_, Ru/SPS or RB/SPS-induced hydrogels. HRP, horseradish peroxidase; Ru, tris (2,2-bipyridyl) dichlororuthenium (II) hexahydrate; SPS, sodium persulfate; RB, riboflavin; (**D**) lithium phenyl-2,4,6-trimethylbenzoylphosphinate (LAP)-induced hydrogel.

Glutaraldehyde is capable of reacting with the side groups of amino acids in protein chains. In SF, for instance, it targets the amino or phenolic hydroxyl groups on the specific amino acids (such as lysine or tyrosine) [69, 70], as partially shown in Figure 3A. For instance, Zhang *et al.* [71] have successfully prepared several kinds of SF-based hydrogel films by using glutaraldehyde as a cross-linking agent (4 µL glutaraldehyde was introduced into 2 mL of 4% SF solution). In this report, the curcumin- and glycerol-contained SF films presented desired antibacterial function and suitable flexibility and good water and gas permeability. Based on this crosslinking agent, Batra and Purwar [72] have also prepared a kind of *Antheraea mylitta* SF/gelatin hydrogel with high water uptake capacity and cytocompatibility.

Genipin, a natural crosslinking agent, possesses a fused bicyclic structure of pentane and dihydropyran rings. It functions by covalently bonding to primary amine structures [73, 74]. This kind of crosslinking agent can covalently bond to the amino group of lysine in the SF molecules to form corresponding crosslinking networks, as illustrated in Figure 3B. For instance, Chirila *et al.* [74] have successfully prepared SFH by crosslinking with genipin. Specifically, the SF and genipin solutions were thoroughly mixed and then held at 40°C until the sample gelled. During this process, the mixture gradually turned blue or bluish-violet—a recognized indicator of genipin reacting with amino compounds. Heat treatment at 37°C (body temperature) for a minimum of 24 h could also facilitate the chemical crosslinking of adjacent SF molecules by the activated genipin [73]. In addition, the SF/gelatin composite hydrogel could also be fabricated by the crosslinking with genipin, and this kind of hydrogel could well maintain the cells’ viability and proliferation [75].

It’s worth noting that the sol-gel transition process is relatively long (at least a few hours), and the small molecule crosslinking agents may exert specific toxicity on cells. Thus, thoroughly removing the non-crosslinked small molecules in the hydrogel is quite important. Relatively, as a natural compound, genipin is observed to be much less toxic than glutaraldehyde, thus it is more widely used as a corresponding small crosslinking agent in the biomedical field. In this field, some other harmless agents, such as citric acid, have also been tried to prepare similar protein hydrogels [76].

#### Crosslinked through the functional group of phenolic hydroxyls

The phenolic hydroxyl in the tyrosine of SF molecules is another important functional group for the formation of chemical crosslinking networks. The core principle of this strategy is the formation of a di-tyrosine bond among SF molecules based on this functional group [34]. In this field, the commonly used enzyme catalysis and initiator are horseradish peroxidase (HRP) [77], tris (2,2-bipyridyl) dichlororuthenium (II) hexahydrate (Ru) [78] and riboflavin (vitamin B2, RB) [79], as schematically summarized in Figure 3C.

HRP is usually accompanied by H_2_O_2_ to initiate phenolic hydroxyl to form di-tyrosine bonds, and the optimal working temperature is body temperature, which is quite suitable for live cell encapsulation. For example, by employing extrusion-based low-temperature 3D printing, Li *et al.* [52] fabricated a macroporous hydrogel scaffold. The hydrogel is based on SF and tyramine-modified gelatin crosslinked via the HRP/H_2_O_2_ system. Studies have further shown that combining this hydrogel scaffold with stem cell aggregates offers considerable promise for cartilage regeneration. A similar hydrogel synthesis strategy has also been applied by Ziadlou *et al.* [80] to fabricate SF-based hydrogels for drug delivery and cartilage repair.

Generally, the gelation time of the HRP system for a pure SF solution is relatively longer, usually more than 20–30 min. The further developed Ru system could be quicker for the related gelation, usually in less than a few minutes. As for the Ru system, it is commonly accompanied by ammonium persulfate (APS) or sodium persulfate (SPS) to initiate phenolic hydroxyl to form di-tyrosine bonds under the excitation of almost lossless white light. The excitation condition and the substance used in this strategy were also mild and suitable for live cell encapsulation and 3D cell culture. For example, using the solution combined with SF and butyl glycidyl ether (BGE) as raw material, Bae *et al.* [81] have chosen this Ru/SPS initiation system to fabricate a chemically crosslinked SF/BGE hydrogel, which provides a suitable microenvironment for enhancing fibroblast adhesion and proliferation. Just because of the feasible fabrication and mild gelation process, Zhu *et al.* [25] have also applied this strategy to successfully fabricate five kinds of SFHs that possessed equivalent initial properties yet degraded at different rates, with the aim of investigating how hydrogel degradation influences encapsulated stem cell proliferation and differentiation.

RB is a natural material with almost no cytotoxicity. It is usually accompanied by SPS or H_2_O_2_ to initiate phenolic hydroxyl to form di-tyrosine bonds under the excitation of white light or ultraviolet (UV) light. In 2016, this novel strategy to photocrosslink SF was reported by Applegate *et al.* [79]. This report also clearly determined the mechanism of corresponding gelation. A few years later, Piluso *et al.* [82] achieved rapid gelation of cell-encapsulated SFHs by employing visible light-induced crosslinking with RB and SPS. It has been proved that the biocompatible RB, combined with SPS and visible light induction, is highly feasible for live cell encapsulation and 3D cell culture.

These three kinds of strategies have been popularly used for the preparation of chemically crosslinked SFHs using the natural SF source. Relatively, the gelation time of the Ru system (less than a few minutes) is significantly shorter than that of the HRP system (more than 20–30 min), and the gelation time of the RB system is quite similar to that of the Ru system. In addition, some other enzymes such as tyrosinase, glutamine transferase and laccase have also been attempted to fabricate the SFH based on the crosslinking points of di-tyrosine bonds [83–85].

#### Crosslinked through the functional group of carbon-carbon double bonds

To enable SF for digital light processing (DLP) 3D printing, scientists have developed a new kind of chemically modified SF, which owns the unique functional group of “C = C”. Under UV light excitation (about 405 nm in wavelength) and the initiation of LAP, this functional group enables rapid crosslinking of the modified SF, thereby serving as an important bio-ink for DLP 3D printing and an effective strategy for fabricating chemically crosslinked SFHs.

In this field, Kim *et al.* [86] have used glycidyl methacrylate (GMA) to modify SF at about 60°C conditions and finally obtained the methylacrylated silk fibroin (SFMA), which contained sufficient carbon-carbon double bonds. Then, they used this kind of bio-ink and DLP 3D printing to fabricate various hydrogels with different shapes. Demonstrating its versatility, the SFMA bioink has been used to fabricate complex organ constructs—such as the ear, brain, heart and blood vessels—all of which show high shape fidelity and excellent biocompatibility. Based on this approach, numerous studies have utilized such modified SF to fabricate SFHs, which use carbon-carbon double bonds as the hydrogel-forming crosslinkable groups, as illustrated in Figure 3D. For instance, Hong *et al.* [51] have constructed a complex rabbit trachea-shaped SFH scaffold encapsulated with a large number of chondrocytes by using SFMA. Results showed that the *in vivo* transplantation with chondrocyte-laden SFHs enabled the production of functional and efficient engineered cartilage. These studies promised the fabrication of SFHs with excellent biocompatibility and functionality by using the functional group of carbon-carbon double bonds.

It needs to be noted that the above-mentioned contents are mostly focused on the fabrication of hydrogels with a pure SF component. For considering the complementary usage of different material properties and the utilization of better function and mechanical properties of multiple cross-linking networks, interpenetrating polymer network hydrogels based on SF and other related polymers have also been developed for the corresponding biomedical applications [87–89]. In addition, only the strategies that hold a mild gelation process with no obvious toxicity or harmlessness to living cells are suitable for cell encapsulation and 3D cell culture studies. Furthermore, the relevant chemical crosslinking mechanisms indicate that the majority of chemically crosslinked hydrogels form networks among specific amino acid residues in the hydrophilic segments of SF molecules. Thus, in theory, this interaction does not significantly alter the intrinsic conformational transition tendency and potential of SF molecules within these hydrogels.

## The protein conformational transition within SFHs

The inherent structures of chemically crosslinked SFHs have generally exhibited better stability and mechanical strength compared to the physically crosslinked ones. In addition, the adjustable and larger pore structures of chemically crosslinked SFHs are also more suitable for 3D cell culture. So, the chemically crosslinked SFHs showed more biomedical application potential, which has consequently led to their widespread adoption in the domains of tissue engineering and 3D cell culture. Herein, the mentioned protein conformational transition just happened within these widely used chemically crosslinked SFHs.

### Intrinsic mechanism of the protein conformational transition within SFHs

Natural SF exhibits a distinctive secondary structure characterized by alternating hydrophobic and hydrophilic chain segments. These hydrophobic segments, predominantly comprising repetitive sequences [e.g. (-GAGAGS-)_n_ or (-GAGAGY-)_n_], possess a high propensity for self-assembly, thereby forming stable *β*-sheet crystalline structures (see Figure 2A). During the natural spinning process, the inherent alternation of hydrophobic and hydrophilic domains drives the spontaneous self-assembly of a distinctive architecture [36, 61]. This structure consists of regularly alternating amorphous random coil regions and crystalline *β*-sheet regions, which is largely responsible for the exceptional mechanical strength and toughness of natural silk.

It should also be noted that the biophysical mechanisms governing the conformational transition of protein molecules into *β*-sheets and other higher-order structures are known to depend not only on the primary amino acid sequence but also on the solvent environment. For instance, salt ions can remove water molecules from the hydrated surface of SF, thereby promoting a conformational transition from random coil to *β*-sheet [59]. Similarly, exposure to methanol can trigger structural changes by interacting with the polar groups of SF macromolecules, influencing hydrogen bonding between protein chains and ultimately inducing the formation of *β*-sheet structures [90]. As for the conformation transition kinetics, it has been reported that the transition induced by ethanol, whether in film or solution state, occurs through three distinct stages [91, 92]. This process begins with the nucleation of *β*-sheet structures, followed by a rapid phase of *β*-sheet growth. The final stage involves a slower process that refines and perfects the initially formed *β*-sheet structures [91].

The formation of most chemically crosslinked SFHs is achieved through cross-linking reactions that target active functional groups on amino acids within the hydrophilic segments [34, 44]. A key outcome of this synthetic route is the preservation of the hydrophobic chain segments in an “intact” state. In accordance with the intrinsic features of natural SF molecules (Figure 2A), these preserved hydrophobic segments can freely move and self-assemble. This process inevitably induces a conformational transition in the SF molecules from random coils to the more thermodynamically stable *β*-sheet structures [54, 55], as schematically shown in Figure 1A. This mechanism fundamentally explains the protein conformational transitions that occur within typical chemically crosslinked SFHs, both under *in vivo* implantation and *in vitro* cell culture conditions. Based on this mechanistic analysis, we can further summarize some essential prerequisites for SFHs more likely to undergo the corresponding conformational transition, such as (1) the SF molecular chain should be as long as possible, (2) the presence of freely movable hydrophobic chain segments with sufficient length and (3) the undamaged and unchanged amino acid sequence of the natural hydrophobic chain segments. These are exactly the reasons why the hydrogel of SFMA (a kind of side group-modified SF) could also undergo obvious protein conformational transition [93].

### Characterization of the corresponding transition process

It has been observed that conformational transition of proteins in SFHs is a frequent phenomenon in the settings of both *in vitro* cell culture and *in vivo* implantation. During the related transition process, a higher *β*-sheet content enhances light scattering within the SFH, thereby compromising its transparency. To clarify the corresponding transition process, hydrogel samples can be taken out at predetermined time points for characterizing the changes in *β*-sheet contents, macroscopic transparency states and indicated light transmittance. The corresponding transition rates within SFHs can be comprehensively evaluated by monitoring the rate of change of these material features.

To directly characterize the *β*-sheet contents, the hydrogels were first retrieved and freeze-dried. After lyophilization, the samples were then analyzed using an FT-IR spectrometer. The *β*-sheet content was then determined by calculating the ratio of the indicated peak area (1616–1637 cm^−1^) to the total band area between 1600–1700 cm^−1^, as described in previous studies [25, 54]. Besides, thioflavin T staining is also a feasible and effective strategy to characterize the relative *β*-sheet contents. Specifically, related hydrogels could be stained using dye thioflavin T solution, followed by a rinse with ethanol solution and deionized water. The stained samples need further examination under a microscope capable of transmitted and fluorescence illumination, with fluorescence imaging performed at excitation/emission wavelengths of 495/515 nm. The relative *β*-sheet content can be further evaluated based on the density of fluorescent dots in the micrographs, with higher density indicating higher content [94].

For the transparency changes during the conformation transition process of the hydrogel. The absorbance optical density (OD) value during the transition process could be quantitatively tested by a microplate reader. Changes in OD values, which correlate with variations in light transmittance of the hydrogels, can thus serve as an indirect indicator of the protein conformational transition rates [54, 55]. In addition, the macroscopic transparency of the hydrogels can be easily captured and recorded by a camera, which can also be used to evaluate changes of hydrogels’ transparency, thereby indirectly and simply reflecting the relevant conformational transition process [54, 55].

### Important material characteristic changes induced by the conformational transition

**Figure 4:**
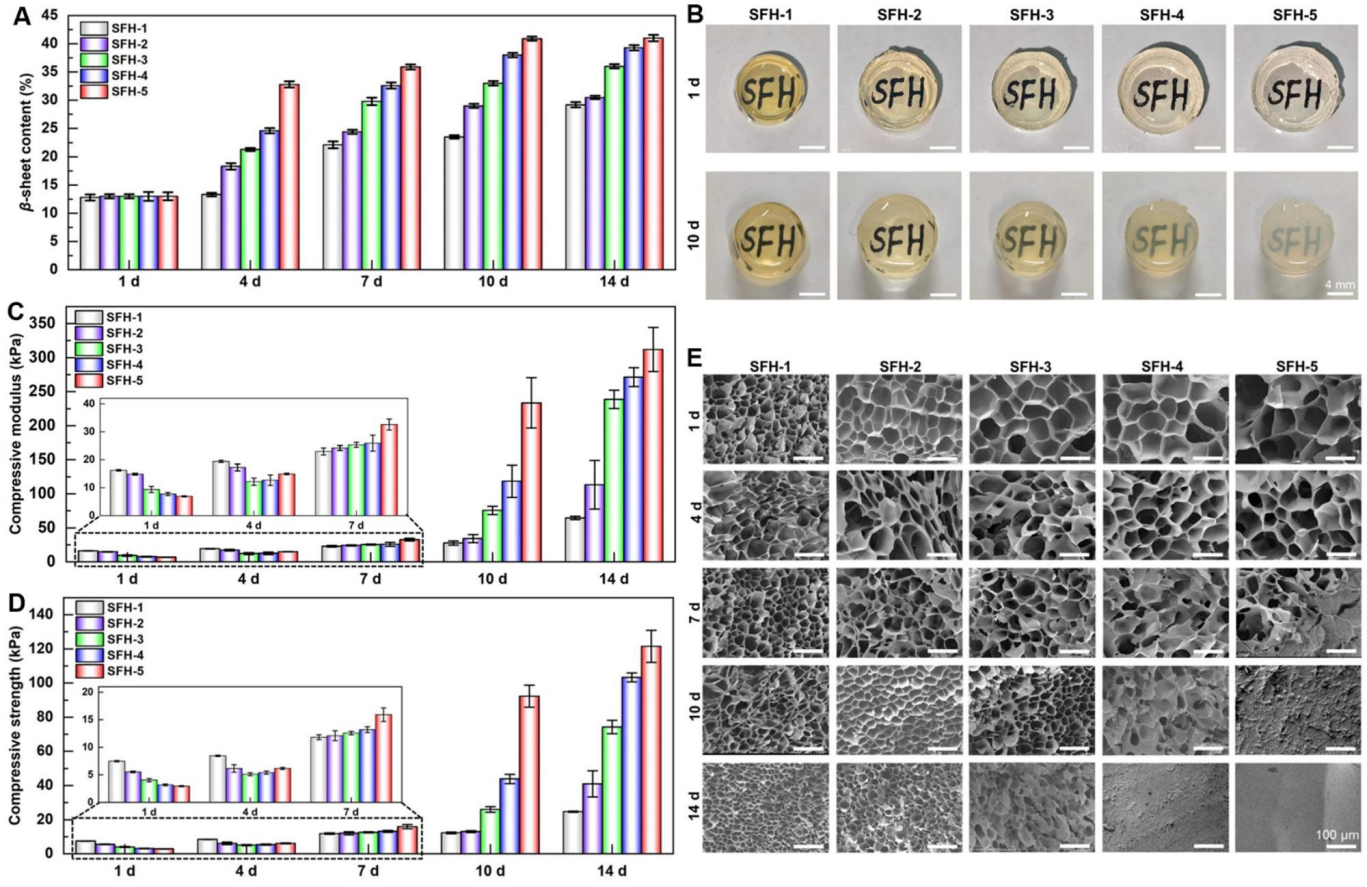
Typical material feature changes induced by the protein conformational transition in SFHs associated with varied initial crosslinking densities. The conformational transition rate increases in the following order: SFH-1 < SFH-2 < SFH-3 < SFH-4 < SFH-5. (**A**) Changes of the *β*-sheet contents; (**B**) changes of the macrotransparency states; (**C**) changes of the compressive modulus; (**D**) changes of the compressive strength; (**E**) changes of the internal pore structures. Images (A–E) were modified with permission [54]. Copyright 2025, Oxford University Press.

More specifically, the transition process intuitively leads to the hydrogel’s change from an initial transparent state with rich random coil structures to a later turbid and opaque state with more *β*-sheet structures. The rate of conformational transition positively correlates with the extent of change in both *β*-sheet content and macroscopic transparency; a faster transition leads to more substantial alterations in these properties (see Figure 4A and B), as reported in our previous report [54]. In this literature, the conformational transition rate increases across the series in the following order: SFH-1 < SFH-2 < SFH-3 < SFH-4 < SFH-5. Moreover, the compressive modulus and strength (see Figure 4C and D) of the hydrogels both gradually increased, while the pore size and porosity gradually decreased during the conformational transition process (see Figure 4E) [54].

Summarily, from a macroperspective, a dynamic material hardening/shrinkage process occurred. All these material features display a positive correlation between their rates of change and the protein conformational transition rates in the SFHs. From the perspective of change trends in material features, the dynamic material microenvironments induced by conformational transitions are quite different from the dynamic microenvironments triggered by the widely studied material degradation.

### Regulation of the CTMs in SFHs

As for a new promising dynamic material microenvironment, how to effectively regulate the mentioned conformational transition is pivotal for its systematic investigation and further applications. Very recently, some pioneer reports [54, 55, 57] have made valuable attempts and explorations in this field. Until now, the developed regulation strategies mainly contain four categories: (1) adding other co-crosslinking components with varied contents within the SF-based composite hydrogels, (2) changing the crosslinking density of the hydrogels, (3) changing the uniformity degree of the crosslinking points of the hydrogels and (4) applying proper external stimuli to the hydrogels.

**Figure 5:**
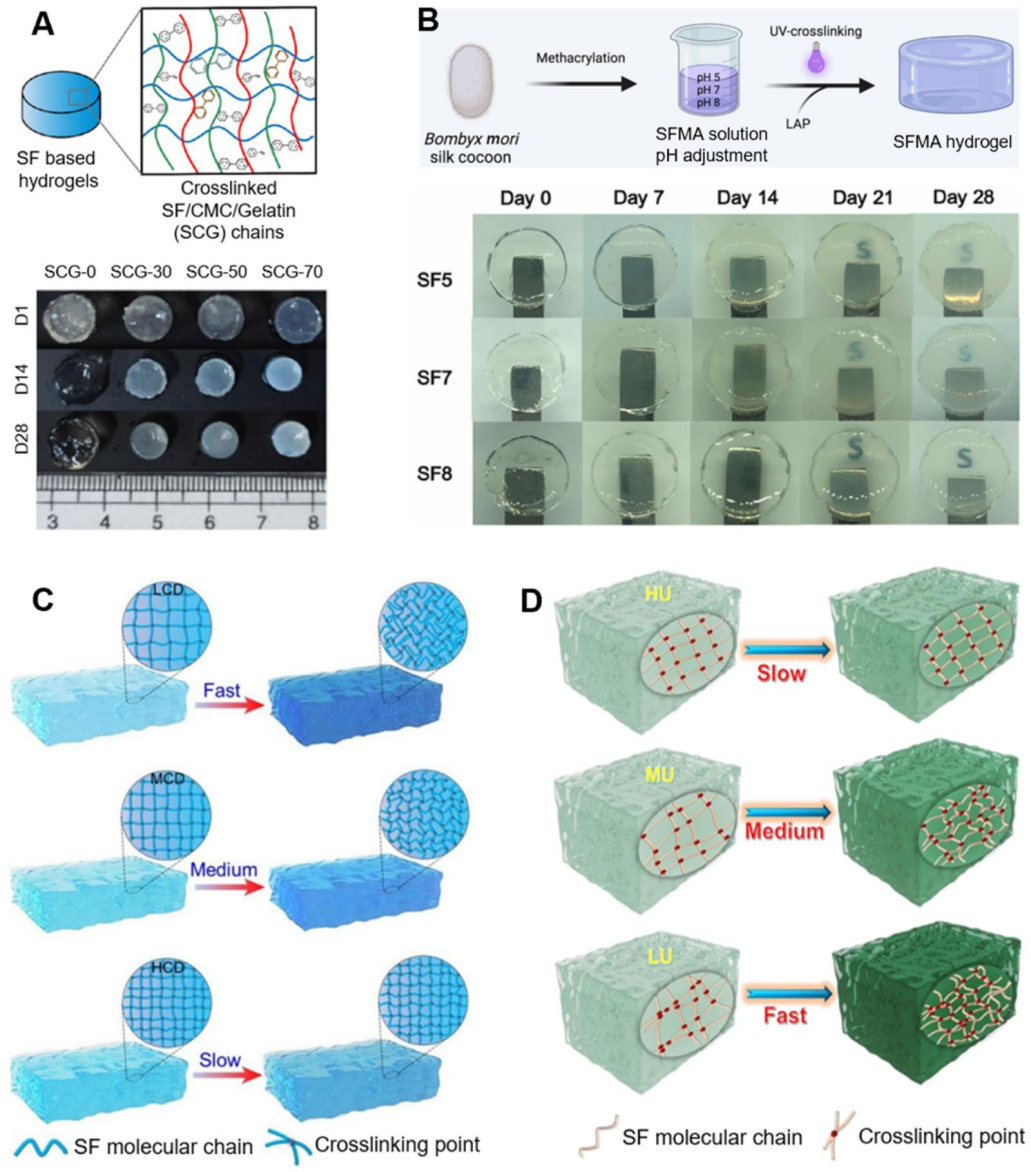
Strategies for the engineering of protein conformational transition in the SF-based hydrogels. (**A**) Changing the ratios of other co-crosslinking components within the SF-based composite hydrogels. CMC: carboxymethyl cellulose, SCG-0: SCG hydrogel with 0% SF, SCG-30: SCG hydrogel with 30% SF, SCG-50: SCG hydrogel with 50% SF, SCG-70: SCG hydrogel with 70% SF; (**B**) changing the pH of hydrogel precursor solution. The fabricated SFMA hydrogels were named SFMA pH 5 (SF5), SFMA pH 7 (SF7) and SFMA pH 8 (SF8); (**C**) changing the hydrogel’s chemical crosslinking density. LCD: low chemical crosslinking density, MCD: medium chemical crosslinking density, HCD: high chemical crosslinking density; (**D**) changing the uniformity degree of the chemical crosslinking points within SFHs. HU: high degree of uniformity, MU: medium degree of uniformity, LU: low degree of uniformity. Image (A) was modified with permission [57]. Copyright 2022, American Chemical Society. Image (B) was modified with permission [93]. Copyright 2021, American Chemical Society. Image (C) was modified with permission [54]. Copyright 2025, Oxford University Press. Image (D) was modified with permission [55]. Copyright 2025, Oxford University Press.

In the related regulation field, the earlier strategy is adding other co-crosslinking components to change the SF ratio within the composite hydrogels [57, 80]. At a lower SF ratio in the composite hydrogel, fewer molecular chains can participate in conformational transition and molecular chains of other components within the hydrogel system further hinder the movement and self-assembly of SF molecules, thus inducing slower and milder conformational transition, and *vice versa*. Under this guidance, Mahajan *et al.* [57] prepared a series of SF-based hydrogels with tunable conformational transition rates through incorporating gelatin and carboxymethyl cellulose (CMC), which are named SF/CMC/Gelatin (SCG) hydrogels. It reported that the group with a lower proportion of SF showed a slower conformational transition rate, and the group with a higher proportion of SF showed a faster transition rate (more quickly became opaque), as briefly illustrated in Figure 5A. In addition, Ziadlou *et al.* [80] have demonstrated that in the hyaluronic acid-tyramine (HA-Tyr)/SF composite hydrogels, the conformational transition of the SF component could enhance the mechanical properties of the hydrogel. And the addition amounts of HA-Tyr could also be used to regulate the conformational transition of the composite hydrogel. Consequently, with the aid of significant cartilage matrix deposition of the encapsulated cells, the composite hydrogel group HA-Tyr20/SF80 (20% HA-Tyr, 80% SF) exhibited a continuous increase in compressive modulus during the 4 weeks of culture. Except for the conformational transition rate, it is important to consider that this strategy also introduces concurrent variations in the hydrogel’s chemical components and initial mechanical properties [57].

In addition, the research by Barroso *et al.* [93] has even proved that the pH of the precursor solution could also be used to regulate the conformational transition of SFMA hydrogels. More specifically, they fabricated three kinds of SFMA hydrogels named SFMA pH 5 (SF5), SFMA pH 7 (SF7) and SFMA pH 8 (SF8). After culture under physiological conditions, the hydrogels underwent spontaneous conformational transition, characterized by an increase in *β*-sheet content, enhanced mechanical properties and decreased transparency. And finally found that the SF5 showed the fastest conformational transition and the SF8 showed the slowest transition [93], as shown in Figure 5B.

By varying the hydrogel’s crosslinking density, we can effectively modulate the length of freely movable SF chain segments within the network. Under lower crosslinking density conditions, longer freely movable chain segments induce faster protein conformational transition due to their more feasible free movement and self-assembly, and *vice versa*. This in turn allows for effectively adjusting the protein conformational transition rates in the SFHs, as illustrated in Figure 5C. For example, Cai *et al.* [54] have set an optimal ratio of HRP to H_2_O_2_ as 50 U/mL: 12.9 mM (concentrations in the hydrogel precursor solution) for the fabrication of hydrogels. To achieve tunable crosslinking densities, five distinct SFHs were fabricated by maintaining a fixed SF concentration and systematically varying the HRP/H_2_O_2_ concentration in a controlled manner. And the transition rate of protein conformation within the related SFHs generally decreased with the increasing of hydrogel crosslinking density [54].

In about 2020, an interesting report by Cui *et al.* [46] clearly indicated that the mechanical properties of the SFH induced by the Ru/SPS system are more stable during the incubation process, with the underlying reason being that the protein conformational transition rate in the hydrogel induced by the Ru/SPS system is significantly slower than that in the hydrogel induced by the HRP/H_2_O_2_ system. Inspired by this pioneer report, a unique strategy by regulating the uniformity degree of a hydrogel’s chemical crosslinking points has also been proposed for constructing SFHs’ own nearly equivalent initial features but varied transition rates of protein conformation. Compared to hydrogels with high crosslinking point uniformity (HU), those with low uniformity (LU) at a similar overall crosslinking density are reported to contain “longer” hydrophobic segments. The presence of these segments promotes greater mobility and self-assembly, consequently leading to faster conformational transition rates in LU hydrogels [55], as schematically illustrated in Figure 5D. Moreover, maintaining a similar overall crosslinking density across SFHs was essential to ensure comparable initial material features. Guided by relevant theories, Zhao *et al.* [55] selected these two induction systems (Ru/SPS and HRP/H_2_O_2_) based on a shared crosslinking mechanism to prepare SFHs with tunable conformational transition rates. Both rely on di-tyrosine bond formation to build the hydrogel crosslinking network [34]. A key distinction between these induction systems is the molecular weight difference between HRP and Ru (HRP at ∼44 kDa vs. Ru at ∼0.75 kDa) [46]. Furthermore, the Ru/SPS system reacts significantly quicker than the HRP/H_2_O_2_ system, with gelation times of less than a few minutes vs about 30 min, respectively. Under similar processing conditions, the much smaller Ru achieves easier and more uniform dispersion in the hydrogel precursor, which also allows much more rapid crosslinking. This disparity ultimately results in a more uniform crosslinking network for the Ru/SPS system. By combining the Ru/SPS and HRP/H_2_O_2_ systems and tuning their ratios, Zhao *et al.* [55] first constructed varied SFHs with similar overall crosslinking density but differing crosslinking uniformity. It demonstrated an inverse correlation between the protein conformational transition rate and the crosslinking point uniformity [55]. These findings further proved that the control of conformational transition rates could be successfully obtained by modulating the network homogeneity of SFHs.

In addition, it has been reported that applying proper external stimuli to the hydrogels could also be used to regulate the conformational transition of SF biomaterials. For example, Mu *et al.* [59, 95] have applied a proper aqueous salt bath (ionic environment) to rapidly accelerate the SF molecular assembly (conformational transition) to make it insoluble, thus achieving well extrusion-based 3D printing using concentrated SF solutions as inks. The methanol or ethanol solution-treatment strategies have also been reported to effectively enhance the conformational transition of SF molecules in hydrogels. And some unique functional SF materials with excellent mechanical properties or even with spatially controlled conformations could be fabricated based on these strategies [58, 96]. Wang *et al.* [90] have even used water vapor (water molecule involvement) or methanol vapor-induced conformational transition to construct a micropatterned surface on SF films. It should be noted that, although the majority of external stimuli strategies might not be suitable for cell encapsulation studies, they have provided valuable references for the construction of protein-based functional materials.

## Biomedical applications of the CTMs in SFHs

### The regulation of cell proliferation

In 2022, Mahajan *et al.* have developed a triple-network hydrogel system (SCG hydrogel) composed of SF, gelatin and CMC. As a result of the conformational transition of SF molecules, the SCG composite hydrogel exhibited a concurrent increase in stiffness and contraction over time. With higher contents of SF (lower contents of CMC and gelation) in the composite hydrogel, the protein conformational transition is more obvious and faster [57]. Through the live/dead staining and lactate dehydrogenase (LDH) release evaluation results, encapsulated mesenchymal stem cell-derived from the infrapatellar fat pad (IFP-MSCs) behaviors illustrated that the hydrogels with relatively slower conformational transitions (without or lower contents of SF) are relatively more favorable for cell proliferation than the hydrogels with faster conformational transitions. However, except for the varied conformational transition rates, it should be noted that these composite hydrogels differed considerably in their chemical compositions and initial mechanical features in the literature [57]. Accordingly, this report has only roughly reported the comprehensive effects of the conformational transition, chemical composition and initial mechanical properties of the hydrogels on cell proliferation.

**Figure 6:**
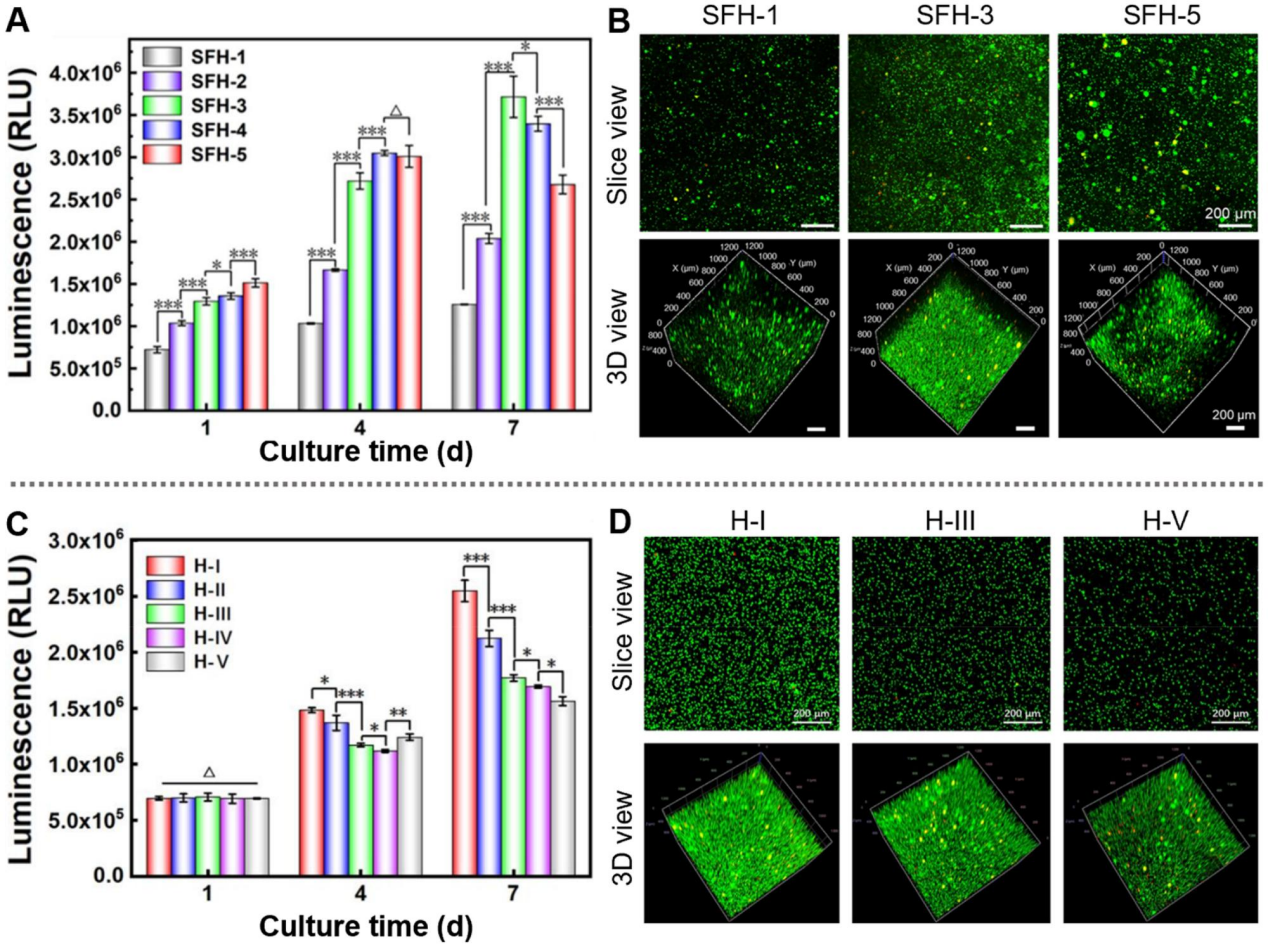
Influence of the CTMs in SFHs on stem cell proliferation. (**A**, **B**) The combined impacts of protein conformational transition and crosslinking density cues. Total cell viability changes (A) and the fluorescent images of the live/dead staining of encapsulated MSCs (B) after indicated culture times; (**C**, **D**) protein conformational transition cues effects. Total cell viability changes (C) and the fluorescent images of the live/dead staining of encapsulated MSCs (D) after indicated culture times. Images (A, B) were modified with permission [54]. Copyright 2025, Oxford University Press. Images (B–D) were modified with permission [55]. Copyright 2025, Oxford University Press.

To effectively eliminate the interference of chemical composition, Cai *et al.* [54] have further fabricated five distinct SFHs with varying conformational transition rates based on modulating their crosslinking densities. These hydrogels, designated as SFH-1 through SFH-5, exhibit a progressive increase in conformational transition rate from slowest (SFH-1) to fastest (SFH-5). They then systematically investigated how the conformational transition of proteins combined with the corresponding crosslinking density cues influences the encapsulated cell proliferation. The cells studied in this literature are bone marrow mesenchymal stem cells (MSCs). In this report, the chemical compositions are all SF within these hydrogels. It was ultimately revealed that a moderate conformational transition rate optimally promoted cell proliferation, whereas transition rates that were too high or too low resulted in slower cell proliferation (see Figure 6A and B). The reported influence on cell proliferation was likely due to the combined effects of the dynamic conformational transition together with the initial crosslinking density within the corresponding hydrogels in this literature [54].

More interestingly, to further minimize the potential interference from differing initial hydrogel properties, a series of SFHs were fabricated by Zhao *et al.* that exhibited varied conformational transition rates yet possessed identical initial characteristics (average pore size and compressive mechanical properties) [55]. The conformational transition rate increases across the series in the following order: H-I < H-II < H-III < H-IV < H-V. Then, they pioneered the study and uncovered the impacts of independent CTMs on encapsulated cell proliferation when eliminating the interference factors as much as possible. It showed that all SFHs supported the proliferation of encapsulated MSCs to varying degrees, with a relatively slower conformational transition rate proving more conducive to enhancing cell proliferation (see Figure 6C and D) [55].

Overall, these studies have clearly demonstrated and confirmed that the dynamical microenvironment induced by protein conformational transitions can significantly influence encapsulated cell proliferation, with a relatively slower transition rate proving more favorable for cell proliferation. That’s probably because the maintenance duration of the relatively large pore size state of the hydrogel was longer in the group with slower conformational transitions, and the large pore size facilitates material exchange, thereby being beneficial for cell proliferation. While it needs to be noted that in the aforementioned studies, the initial crosslinking density features of the hydrogel, accompanied by different initial pore sizes and modulus, can also significantly influence cell proliferation. Thus, under the combined effects of the dynamic conformational transition together with the initial crosslinking density, the hydrogels with moderate initial crosslinking density and conformational transition rate optimally promoted cell proliferation (see Figure 6A). These results offer valuable references for the development and application of SF-based hydrogels in 3D cell culture and other biomedical fields.

### The regulation of cell lineage commitment and phenotype maintenance

Based on the typical studies on cell-material interactions [2, 3], the prominent stiffening/shrinkage properties accompanied by the conformational transition within SFHs might be beneficial for cell chondrogenesis. In addition, the SFHs have also been reported to have great application potential for cartilage repair [21, 25, 37]. Therefore, majority reports on regulation of cell lineage commitment in this field are focused on chondrogenic differentiation.

**Figure 7:**
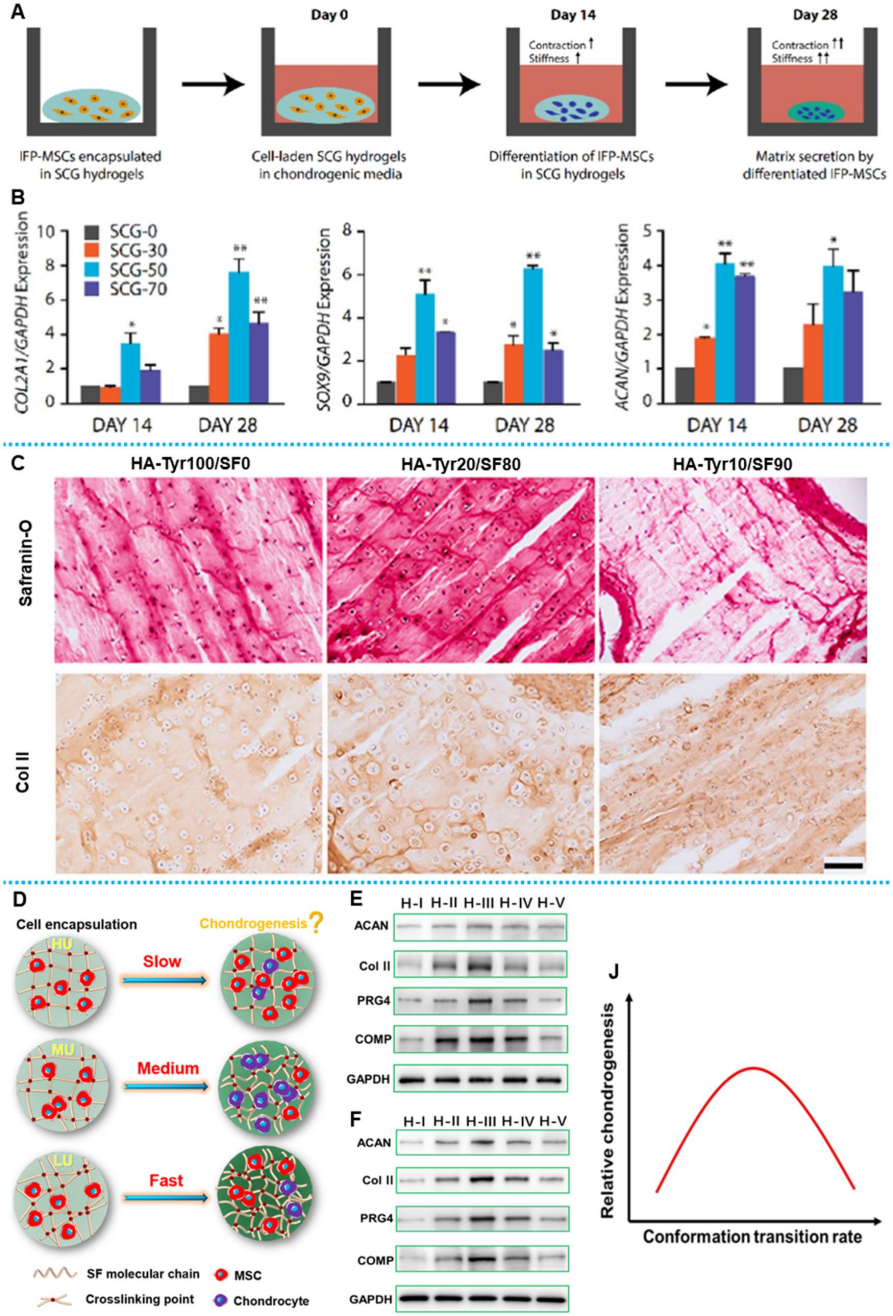
Influence of the CTMs in SFHs on stem cell chondrogenesis and chondrocyte phenotype maintenance. (**A**) Cartoon illustration of the IFP-MSCs cultured within SCG hydrogels to reveal the corresponding conformational transition associated with the initial chemical and mechanical cues’ effects on stem cell chondrogenesis; (**B**) relative cartilage-specific markers (Col II, SOX9 and ACAN) expressions of the cells in (A) after 2 and 4 weeks of *in vitro* chondrogenic induction; (**C**) Safranin-O and Col II staining of the chondrocytes in the composite hydrogels of HA-Tyr100/SF0, HA-Tyr20/SF80 and HA-Tyr 10/SF90 to show the corresponding proteoglycan and Col II depositions (chondrocyte phenotype maintenance) after 3 weeks’ culture. Scale bar, 100 μm. (**D**) Schematic representation to show the potential impacts of the sole CTMs on the stem cell chondrogenesis in hydrogels; (**E**, **F**) relative expression of chondrogenic marker proteins of the cells in (D) after 6 and 12 days of culture, respectively; (**J**) schematic illustrating the chondrogenesis trend vs conformational transition rate. Images (A, B) were modified with permission [57]. Copyright 2022, American Chemical Society, Image (C) were modified with permission [80]. Copyright 2020, Elsevier B.V. Images (**D–J**) were modified with permission [55]. Copyright 2025, Oxford University Press.

Through the evaluation of chondrogenic-specific genes expression, Mahajan *et al.* [57] have found that the SCG composite hydrogels with medium conformational transition rate (SCG-50, with 50% SF) could significantly enhance the chondrogenesis of the encapsulated IFP-MSCs. While the SCG composite hydrogels with slower (SCG-0 and SCG-30, with 0% or 30% SF) or faster (SCG-70, with 70% SF) conformational transition rate presented lower chondrogenic-specific genes expression (see Figure 7A and B). Although different SCG hydrogels presented varied chemical components and mechanical properties in this report, it still hinted that appropriate material stiffening/shrinkage processes induced by the protein conformational transition could enhance the encapsulated cell chondrogenesis to some extent. In addition, Ziadlou *et al.* [80] have also fabricated a series of SF-based composite hydrogels, which are named HA-Tyr/SF hydrogels. Then they encapsulated chondrocytes in the composite hydrogels and further found that the cell-material composite of the group HA-Tyr20/SF80 (20% HA-Tyr, 80% SF) exhibited a continuous increase in compressive modulus during the 4 weeks of culture. More interestingly, the encapsulated chondrocytes in the HA-Tyr20/SF80 with a suitable stiffening/shrinkage process also maintained a better-preserved chondrocyte phenotype when compared to the hydrogels of HA-Tyr100/SF0 and HA-Tyr10/SF90, which showed more obvious proteoglycan and Col II deposition (see Figure 7C).

Furthermore, by fabricating pure SFHs with varied conformational transition rates (achieved *via* adjusting hydrogel crosslinking density), Cai *et al.* [54] systematically investigated the combined influence of protein conformational transition and crosslinking density cues on the encapsulated cell’s chondrogenesis. In this report, the hydrogels were categorized as SFH-1 to SFH-5 in order of increasing conformational transition rate. Results indicated that a moderate transition rate (SFH-3) optimally enhanced stem cell chondrogenesis, whereas transition rates that were either too fast or too slow could slow down this differentiation process. The moderate conformational transition rate is enabled by moderate initial hydrogel crosslinking density in this report. Very recently, Zhao *et al.* [55] even constructed various pure SFHs with matched initial properties but differing in conformational transition rate. This was achieved by adjusting the uniformity of the crosslinking points while maintaining a comparable overall crosslinking density. These resulting hydrogels, designated H-I through H-V, respectively, where the conformational transition rates increased sequentially from H-I (slowest) to H-V (fastest). When eliminating the possible interference induced by different initial chemical and physical hydrogel features, encapsulated MSCs’ responses in this literature further decisively proved that a moderate conformational transition rate could significantly enhance cell chondrogenesis (see Figure 7D–J).

All together, these frontier reports [54, 55, 57] comprehensively studied the effects of CTM on the chondrogenic differentiation of encapsulated stem cells, while using different material strategies to regulate the conformational transition rates. Interestingly, regardless of initial differences in the intrinsic properties of hydrogels, existing findings consistently indicate that a moderate rate of protein conformational transition optimally facilitates cell differentiation. Accordingly, a microenvironment with mild material stiffening or shrinkage induced by an appropriate conformational transition rate can markedly enhance chondrogenesis in stem cells. Notably, this kind of dynamic material effect probably outweighs the influence of the static material cues like chemical composition and initial modulus in these researched systems. The stiffening and shrinkage of materials induced by protein conformational transition dictate that cellular chondrogenic potential increases initially and then decreases as the transition rate rises (see Figure 7B and J). This is probably the reason why the main conclusions of these published studies are generally consistent across different material platform construction strategies. These valuable explorations are expected to afford precious reference for the development and application of advanced SFHs and their tunable CTMs in cartilage tissue engineering and cartilage organoid construction fields.

### The construction of ectopically engineered tissue under *in vivo* conditions

**Figure 8:**
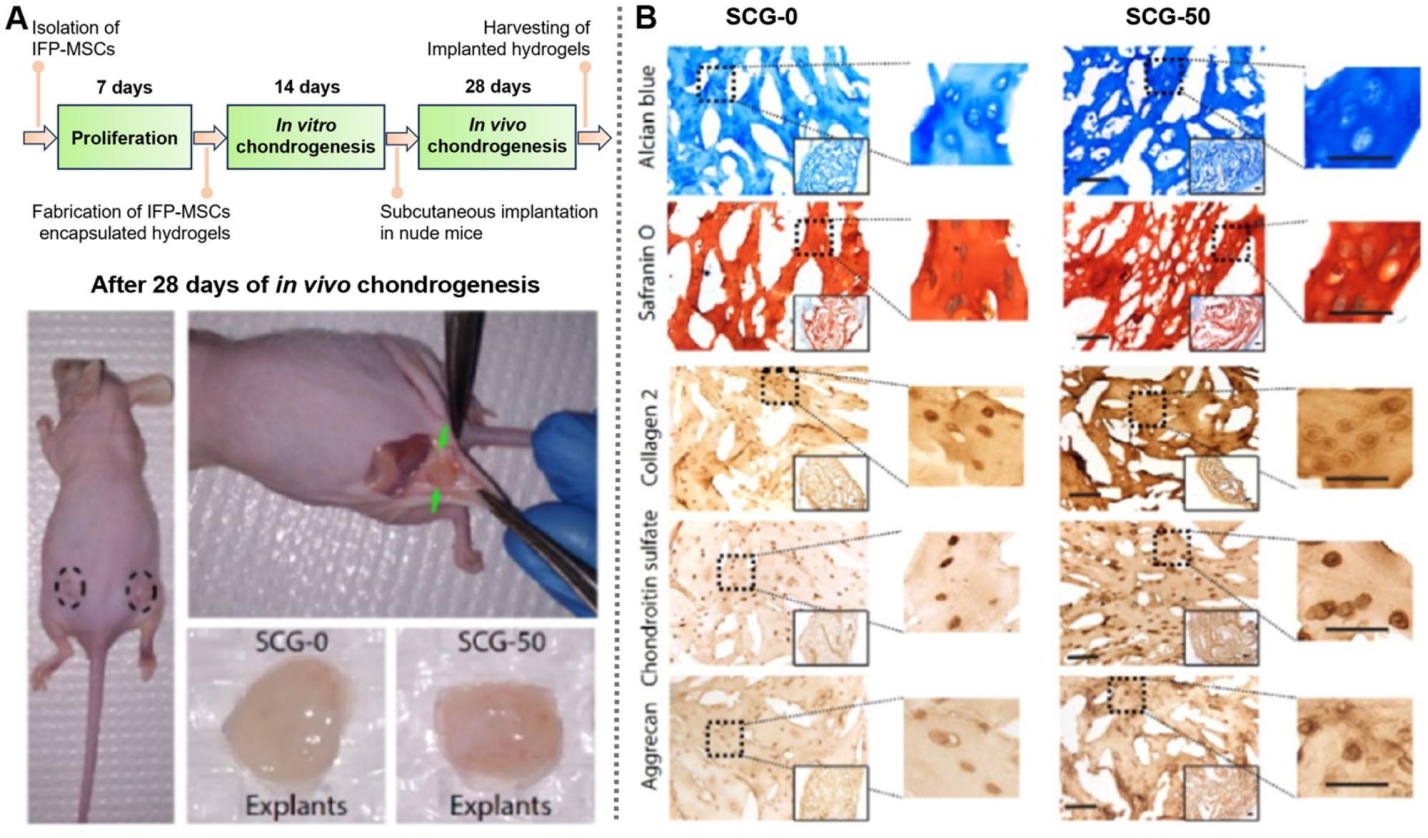
Influence of the CTMs within SFHs on the construction of ectopically engineered cartilage tissue under *in vivo* conditions. (**A**) Up: schematic illustration of the experimental setup for investigating chondrogenesis *in vivo*. Down left: gross photograph of a nude mouse at 28 days post-implantation, showing the separate subcutaneous sites of SCG-0 and SCG-50, each marked with a black dotted circle. Downright: gross photos to show the process of removing implants and the corresponding post explants; (**B**) histological analysis to show the relative cartilage-specific protein expressions of the cells after 28 days of implantation (Alcian blue and safranin O staining for the expression of sGAG, immunohistochemical staining for the expression of Col II, aggrecan and chondroitin sulfate. Scale bars for the normal, inset and high magnification pictures are 100 μm, 200 μm and 50 μm, respectively). Images (A, B) were modified with permission [57]. Copyright 2022, American Chemical Society.

Besides the *in vitro* investigation, Mahajan *et al.* [57] have also first investigated the CTM effects on cell behaviors *in vivo* conditions. More specifically, they have implanted the typical IFP-MSCs contained in composite hydrogels (SCG-0 with no conformational transition and SCG-50 with a medium conformational transition rate) subcutaneous to evaluate the corresponding *in vivo* effects, as schematically shown in Figure 8A. A clear cell morphological difference was noted after 4 weeks of subcutaneous implantation in nude mice: cells in the SCG-50 group presented a spherical shape, while those in the SCG-0 group exhibited a more elongated morphology [57]. Moreover, the cells in the SCG-50 showed obviously higher cartilage ECM deposition, such as the deposition of sGAG, Col II, chondroitin sulfate and aggrecan, as clearly shown in Figure 8B.

In summary, these results indicate that the ectopically engineered tissue formed in the hydrogel SCG-50 with medium conformational transition rate closely resembled native cartilage compared to the hydrogel SCG-0 without CTM, which further confirmed that the induced dynamic material stiffening/shrinkage microenvironment could still play crucial roles in the enhancement of chondrogenesis even under *in vivo* conditions. These important attempts indicated that the SF-based hydrogels with suitable conformation transition microenvironments showed promising potential for cartilage regeneration. In this field, future studies should focus on validating their repair efficacy in realistic cartilage defect models.

### The manipulation of tumor cell growth

**Figure 9:**
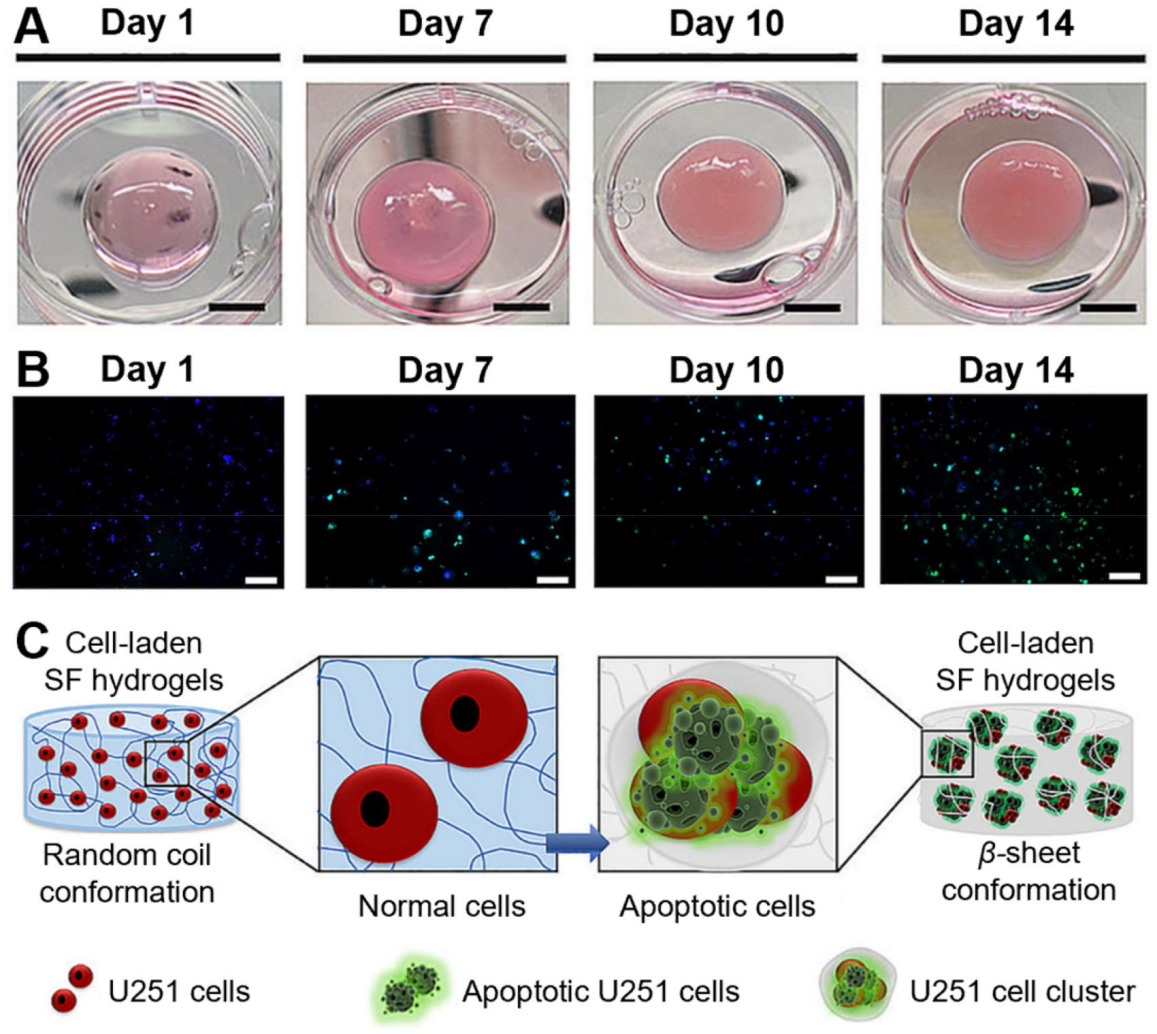
Impacts of the pronounced CTMs on the encapsulated tumor cell growth. (**A**) Macroscopic images of U251 cell-laden SFHs at specified culture time points. Scale bar: 5 mm; (**B**) TUNEL assay staining results of the U251 cells encapsulated SFHs at specified culture time points. Scale bar: 200 μm; (**C**) schematic illustration of the tumor cell apoptosis process induced by the serious conformational transition of SFHs. Image (A) was reproduced with permission [94]. Copyright 2018, Public Library of Science. Images (B, C) were modified with permission [94]. Copyright 2018, Public Library of Science.

Except for the stem cells, scientists have also preliminarily investigated the impacts of obvious CTM on the behaviors of tumor cells. For example, Ribeiro *et al.* [94] have chosen the human neuronal glioblastoma cell line (U251) as a typical tumor cell for investigating cell behaviors under the 3D cell culture conditions (see Figure 9A). Results showed that the initial transparent SFHs enhanced cell proliferation and viability, while during the obvious and serious conformational transition progress, the later *β*-sheet-enriched hydrogels produced partial TUNEL-positive cells (indicating cell apoptosis), as shown in Figure 9B and C. Similar mouse embryonic tumor cell (ATDC-5) behaviors have also been found by Yan *et al.* [97]. In a subsequent *in vivo* study, HeLa cells encapsulated in these SFHs were then implanted into a chick chorioallantoic membrane model [97]. Results showed that hydrogels undergoing pronounced protein conformational transition suppressed both angiogenesis and tumor formation [97].

In summary, the studies in this section indicate that excessive material stiffening/shrinkage microenvironments triggered by severe conformational transition could exert certain negative effects on cell growth and functioning. However, such SFH might serve as a generic injectable system to regulate cancer cell behavior and suppress tumor progression.

These typical literatures about the biomedical applications have comprehensively illustrated that the CTMs could significantly regulate cell behaviors, such as cell growth, proliferation and chondrogenesis. According to the typical reports in the field of cell-material interactions [2, 3] and the material features induced by the conformational transitions, it proposes that this influence is likely determined by a combination of the following factors. (1) Hydrogel pore size: The decreased hydrogel pore size will in turn cause cell contraction and condensation. It has been reported that an appropriate decrease in cell spreading and cell volume favors the chondrogenic differentiation of stem cells [98–100]. However, excessively small cell size may restrict the free proliferation of cells [101]. (2) Hydrogel modulus: The increased hydrogel modulus will bring it from a relatively low modulus closer to the biomimetic requirement modulus of cartilage tissue. Relevant studies indicate that a higher and biomimetic modulus is more conducive to cell proliferation and chondrogenesis [57, 102, 103]. (3) Dynamic stimulation: The material hardening/shrinkage is inherently a dynamic process, thereby providing continuous dynamic contact stimulation to the cells. Studies have shown that dynamic stimuli such as hydrostatic pressure, ultrasound and material degradation can significantly affect cell functions [21, 24, 29]. Based on these more specific factors, the potential signaling pathways through which conformational transitions influence cell behaviors might include the FAK-PI3K-Akt, MAPK and Wnt/*β*-catenin signaling pathways [104–106], which are sensitive to the cell membrane contact stimulations. However, the actual signaling pathway mechanism has not yet been definitively reported and requires further investigation and validation.

## Conclusions and perspectives

Centering on the unique dynamical microenvironment induced by protein conformational transitions within SFHs, this review systematically analyzed the underlying mechanisms governing these transitions and their consequent dynamic material characteristics. Building upon this foundation, we comprehensively summarized advanced strategies for effectively engineering such transitions. Subsequently, frontier research leveraging this dynamic microenvironment is discussed, with a focus on its valuable applications in cell behavior regulation and ectopically engineered tissue construction. While significant advancements have been made, the field of regulating and applying this unique dynamic material microenvironment is still nascent. Consequently, further extensive and in-depth investigations are imperative, particularly in the following key areas.

### Make the controlling of conformational transition more effective and precise

More effective and precise regulation of the conformational transition will make this dynamic material microenvironment more controllable and suitable, thus enhancing its repeatability and usability. On the one hand, the higher molecular weight of SF guarantees its unique natural characteristics, thus making the engineering of the protein conformational transition in SFHs more achievable and effective. So, it is necessary to minimize the damage of SF molecules when extracting them through the degumming and dissolution processes [107]. On the other hand, narrow molecular weight distribution makes the hydrogel networks more homogenous, thus making the regulation of protein conformational transition more controllable and precise. Therefore, extraction of SF molecules with higher molecular weight and narrow molecular weight distribution can probably make the related control more effective and precise. To further develop a related simulation model for predicting the conformational transition dynamics is also an important research topic in this field, which could be useful for providing more in-depth understanding and precise control strategy designing about the conformational transition. Further extensive research is still required to accumulate more experimental data and support the development and training of simulation models. In addition, the development of related novel and controllable hydrogel synthesis strategies or the introduction of on-demand external stimuli to effectively induce and accelerate the corresponding conformational transition might also play important roles in feasibly achieving this goal.

### Further revealing the molecular mechanism of the influence of protein CTMs on cell functions and behaviors

Clarifying the molecular mechanism through which CTMs modulate cell functions and behaviors provides a robust knowledge base and adequate confidence for developing excellent commercial SFH matrices and scaffolds. These materials possess an appropriate CTM, enabling effective cell fate control and enhanced tissue defect repair. In addition, the revealed molecular mechanism also deepens the understanding of the corresponding working pathway and further promotes the development of other collaborative strategies to more efficiently utilize this unique and promising dynamic material microenvironment. According to the essential material features induced by conformational transitions, the potential signaling pathways through which conformational transitions regulate cell behaviors might include the FAK-PI3K-Akt, MAPK and Wnt/*β*-catenin signaling pathways, which are sensitive to the cell membrane contact stimulations. However, the specific signaling pathway mechanism has not yet been definitively reported, which requires further investigation and validation.

### *In vivo* evaluation of the actual effectiveness of protein CTMs on tissue regeneration

Until now, the related material construction and application studies are still in their infancy, and the majority of the application research is still focused on the *in vitro* cell experiments. Accordingly, further *in vivo* evaluations are urgently required in subsequent studies, especially to clarify how this dynamic material microenvironment affects the repair and regeneration of actual tissue defects. In the *in vivo* evaluation, consider the obvious material stiffening/shrinkage phenomenon induced by the conformational transition of proteins. It is recommended to design SFHs with appropriately larger dimensions in advance, based on their corresponding shrinkage rates. Before implantation, the stem cell-encapsulated hydrogels and other control group materials should be pre-cultured *in vitro* for a period of time until the conformational transition has almost completed. This approach aims to better fill the tissue defect site and achieve desired integration with the surrounding normal tissue. Furthermore, the protein CTM of SFHs can also be utilized to facilitate the construction of tissue-engineered cartilage through *in vivo* ectopic implantation (e.g. subcutaneous implantation), followed by the evaluation of cartilage defect repair based on the pre-established tissue-engineered cartilage. Of course, the rate of protein conformational transition in the *in vivo* implantation environment may differ somewhat from that observed in the *in vitro* simulated environments, which can be effectively adjusted and refined according to actual conditions. As for further accelerating its potential application in the human body for disease treatment, some large animal experiments and even clinical trials will also be required in the future.

### Excellent cartilage organoid construction based on the promising protein CTM

As a highly biomimetic 3D cell culture model, ideal organoids possess significant advantages that are unattainable by traditional 2D cell culture and animal model experiments [4, 108–110]. Organoid technologies demonstrate substantial application value across multiple domains, including exploring novel tissue repair strategies and mechanisms, disease modeling, drug screening and investigating *in vivo* mimic cell-material interactions. The mechanical properties of the commonly used materials for organoid construction (such as Matrigel and gelatin) are relatively poor [111, 112]. Consequently, the pioneering constructed cartilage organoids exhibit poor mechanical performance, which is significantly lower than that of natural cartilage and fails to meet the mechanical biomimetic requirements. As for the protein CTM, studies have indicated that appropriate stiffening/shrinkage of the material, driven by conformational transition, could significantly elevate the chondrogenic differentiation of encapsulated cells [54, 55, 57]. Moreover, this unique dynamic process could also obviously elevate the compressive mechanical features of the hydrogel matrix. Based on these useful features, the effective utilization of the dynamic material stiffening/shrinkage process within SFHs is expected to significantly promote and ensure the construction of cartilage organoids with both excellent functional performance and biomimetic mechanical features. Related analysis and prospect research will also provide a useful reference for the construction of other hard tissue organoids.

### Development of SFHs with high transparency and stability under the corresponding theoretical guidance

A few studies have demonstrated that the SFHs showed promising application potential in the fields of contact lenses and artificial corneas [79, 113]. In these unique application fields, how to inhibit the possible protein conformational transition in the related hydrogels becomes an extremely important issue that needs to be addressed. The summarized conformational transition mechanism and regulation strategies in this review have also provided important theoretical guidance and reference for the development of SFHs with high transparency and stability. For example, designing and fabricating SF-based hydrogels with high crosslinking density and high uniformity of crosslinking points could probably obstruct the mentioned conformational transition as much as possible. In addition, suitable modification to the hydrophobic segments of SF molecules or combining SF with other co-crosslinking components might also effectively prevent the formation of *β*-sheet structures, as it will effectively disrupt the formation of such structures.

### Development of functional SF materials as soft as brain or as hard as bone based on relevant knowledge

Theoretically, though, with appropriate regulation of the protein conformational transition process and the regularity arrangement of the crystal structures (*β*-sheet structures) during the fabrication of regenerated SF materials, it could obtain materials with various modulus. Some pioneer researchers have indeed found that through a controllable thermal-assisted film-forming process, a suitable distribution of crystalline and amorphous structures could be formed under this conformational transition process. Through further optimization of this strategy, we finally obtained an ultra-thin and extremely soft SF film (with a Young’s modulus of approximately 30 MPa) [38]. In addition, extremely high-strength SF materials were also fabricated through a high-temperature, combined with ultra-high-pressure molding process. Specifically, SF powder was hot-pressed under this molding condition (about 145°C, 632 MPa) to control the formation of unique crystalline structures (protein conformational transition), which possess a high content of highly orientated *β*-sheet crystal structures. This resulting functional SF material even exhibits a compressive modulus as high as 3 GPa [114]. In the future, it is highly believed that the summarized mechanism and regulation strategies of the conformational transition process within SF materials will provide valuable theoretical guidance and references. These can greatly enhance the controllability of further developing SF functional materials ranging from as soft as a brain to as hard as bone.
